# An inflammation-based prognostic score (mGPS) predicts cancer survival independent of tumour site: a Glasgow Inflammation Outcome Study

**DOI:** 10.1038/sj.bjc.6606087

**Published:** 2011-01-25

**Authors:** M J Proctor, D S Morrison, D Talwar, S M Balmer, D S J O'Reilly, A K Foulis, P G Horgan, D C McMillan

**Affiliations:** 1 University Department of Surgery, Faculty of Medicine-University of Glasgow, Royal Infirmary, Glasgow G31 2ER, UK; 2 West of Scotland Cancer Surveillance Unit, Faculty of Medicine-University of Glasgow, Glasgow G12 8RZ, UK; 3 Department of Clinical Biochemistry, Royal Infirmary, Glasgow G31 2ER, UK; 4 Department of Clinical Pathology, Royal Infirmary, Glasgow G31 2ER, UK

**Keywords:** C-reactive protein, albumin, liver function tests, survival

## Abstract

**Introduction:**

A selective combination of C-reactive protein and albumin (termed the modified Glasgow Prognostic Score, mGPS) has been shown to have prognostic value, independent of tumour stage, in lung, gastrointestinal and renal cancers. It is also of interest that liver function tests such as bilirubin, alkaline phosphatase and *γ*-glutamyl transferase, as well as serum calcium, have also been reported to predict cancer survival. The aim of the present study was to examine the relationship between an inflammation-based prognostic score (mGPS), biochemical parameters, tumour site and survival in a large cohort of patients with cancer.

**Methods:**

Patients (*n*=21 669) who had an incidental blood sample taken between 2000 and 2006 for C-reactive protein, albumin and calcium (and liver function tests where available) and a diagnosis of cancer were identified. Of this group 9608 patients who had an ongoing malignant process were studied (sampled within 2 years before diagnosis). Also a subgroup of 5397 sampled at the time of diagnosis (sampled within 2 months prior to diagnosis) were examined. Cancers were grouped by tumour site in accordance with International Classification of Diseases 10 (ICD 10).

**Results:**

On follow up, there were 6005 (63%) deaths of which 5122 (53%) were cancer deaths. The median time from blood sampling to diagnosis was 1.4 months. Increasing age, male gender and increasing deprivation was associated with a reduced 5-year overall and cancer-specific survival (all *P*<0.001). An elevated mGPS, adjusted calcium, bilirubin, alkaline phosphatase, aspartate transaminase, alanine transaminase and *γ*-glutamyl transferase were associated with a reduced 5-year overall and cancer-specific survival (independent of age, sex and deprivation in all patients sampled), as well as within the time of diagnosis subgroup (all *P*<0.001). An increasing mGPS was predictive of a reduced cancer-specific survival in all cancers (all *P*<0.001).

**Conclusion:**

The results of the present study indicate that the mGPS is a powerful prognostic factor when compared with other biochemical parameters and independent of tumour site in patients with cancer.

Cancer incidence is increasing in the United Kingdom as well as on a global basis (Boyle and Levin, 2008). Over one in three people in the United Kingdom will develop cancer during their lifetime, with around 150 000 people dying each year as a consequence of cancer (Cancer Research UK, 2007, 2008). Such a burden of disease accounts for a significant proportion of annual health care spending in the United Kingdom, United States and worldwide (Bosanquet and Sikora, 2004; Boyle and Levin, 2008).

Although it is recognised that the development of cancer has a genetic basis, there is increasing evidence that the host inflammatory response has an important role in the development and progression of cancer (Coussens and Werb, 2002; Mantovani *et al*, 2008; Colotta *et al*, 2009; McDonald *et al*, 2009; Tenesa *et al*, 2010). In particular the systemic inflammatory response, as evidence by C-reactive protein, has an important role in the progression of a variety of common solid tumours (Roxburgh and McMillan, 2010).

The measurement of the systemic inflammatory response has been subsequently refined using a selective combination of C-reactive protein and albumin (termed the modified Glasgow Prognostic Score, mGPS) and has been shown to have prognostic value, independent of tumour stage, in lung, gastrointestinal and renal cancers (McMillan, 2008, 2009). It is also of interest that liver function tests such as bilirubin (Temme *et al*, 2001), alkaline phosphatase (Tonelli *et al*, 2009) and *γ*-glutamyl transferase (Kazemi-Shirazi *et al*, 2007) as well as serum calcium (Leifsson and Ahren, 1996), have also been reported to predict cancer and non-cancer mortality in large cohort studies.

More recently it has been shown that, in a large cohort study of >200 000 patients (Glasgow Inflammation Outcome Study) that the mGPS is elevated in patients with cancer when compared with those without cancer. Moreover, that there were significant inter-relationships between the above biochemical parameters, including those used to compose the mGPS (Proctor *et al*, 2010). Together with these results, the question is therefore raised whether the mGPS and other routine biochemical parameters have independent prognostic value in patients with cancer and whether this applies across different tumour sites.

The aim of the present study was to examine the relationship between an inflammation-based prognostic score (mGPS), biochemical parameters, tumour site and survival in patients with cancer of the Glasgow Inflammation Outcome Study.

## Materials and methods

### Study design

From a cohort previously described (Proctor *et al*, 2010), patients in the North Glasgow who had a single blood sample taken for C-reactive protein, albumin and calcium, as well as liver function tests where available, and a diagnosis of cancer were considered. Briefly, patients who were sampled incidentally between the 1 January 2000 and the 31 December 2006 were considered and if more than one set of measurements were available for a given patient, only the initial set was used. Only patients with blood samples taken within two years prior to their cancer diagnosis were included. This was done with the premise that they would likely have an ongoing malignant process at the time of sampling. Also, only patients who had complete Cancer Registry follow up (detailed below) were included in the study. Patients were excluded if they were under 16 or did not have a complete set of identifying details (name, date of birth and hospital number).

Cancer diagnosis was established through linkage with the Scottish Cancer Registry using exact matches of patients’ forename, surname and date of birth followed by a Soundex phonetic matching algorithm if initial exact matching was unsuccessful. At the time of data collection, the Scottish Cancer Registry held complete pathological and clinical cancer diagnosis records from 1 January 1980 to 31 December 2006 and mortality follow-up until 30 June 2009. In those who had died, cancer-specific deaths were classified as patients whose primary cause of death matched their primary cancer diagnosis. All other deaths were classed as non-cancer-specific deaths.

Cancers were coded in accordance with the International Classification of Disease 10 (ICD 10) and broadly grouped according to tumour sites: breast, bladder, gynaecological, prostate, gastroesophageal, haematological, renal, colorectal, head and neck, hepatopancreaticobiliary and pulmonary cancer. These groups were listed in order of the magnitude of their inflammatory status as shown previously (Proctor *et al*, 2010). Patients with multiple malignancies, metastatic disease or cancer of an unknown origin were excluded.

The study was approved by the Research Ethics Committee, North Glasgow NHS Trust.

### Methods

Patients with routine laboratory measurements of C-reactive protein, albumin and calcium were obtained by systematically searching the North Glasgow biochemical database system. The limit of detection of C-reactive protein was a concentration of <5 mg l^−1^. The mGPS was constructed, using C-reactive protein and albumin, as follows: patients with both an elevated C-reactive protein (⩾10 mg l^−1^) and low albumin (<35 g l^−1^) were allocated a score of 2; patients in whom only C-reactive protein was elevated (⩾10 mg l^−1^) were allocated a score of 1 and those with a normal C-reactive protein were allocated a score of 0 (McMillan *et al*, 2007). The rationale and basis of the mGPS has been previously described (McMillan *et al*, 2008). Serum C-reactive protein, albumin and liver function tests, including bilirubin, Alk phos, AST, ALT and GGT, as well as calcium adjusted for albumin (Ashby *et al*, 1986), were classified in accordance with the NHS Greater Glasgow and Clyde Biochemistry Laboratory reference ranges.

ICD 10 codes were used to identify the site of cancer diagnosis. These include breast (C50), bladder (C67), gynaecological (C51–58), prostate (C61), gastroesophageal (C15–16), haematological (C81–96), renal (C64–65), colorectal (C18–20), head and neck (C00–14, C30–32), hepatopancreaticobiliary (C22–25) and pulmonary (C34, C45) cancer.

Deprivation was measured with the Scottish Index of Multiple Deprivation (SIMD) 2006 and in this study was presented with the least deprived being scored as 1 to the most deprived scoring 5. The SIMD 2006 classification of deprivation is based on an individual's postcode and is derived from the measurements of 37 indicators across seven domains including income, employment, education, housing, health, crime and geographical access. The SIMD 2006 is the recommended method for the measurement of deprivation in Scotland by the Information Services Division on behalf of NHS Scotland and the Scottish Government Department of Health (Bishop *et al*, 2004).

### Statistical analysis

Survival, overall and cancer-specific, was calculated from the time of cancer diagnosis to death. Cancer groups with <150 cancer-specific deaths were excluded to ensure the statistical power. Analysis was carried on all cancer patients as well as on a subgroup of patients who had a diagnosis of cancer made within 2 months following their blood sample. This was carried out in order to examine the relationships between the mGPS, biochemical parameters and survival in all patients with a likely ongoing malignant process (patients sampled within 2 years before a diagnosis of cancer) and those at the time of diagnosis (patients sampled within 2 months before a diagnosis of cancer).

The mGPS and biochemical proportionality assumptions were explored using log–log plots and were found to be satisfactory. Kaplan–Meier estimator was used to analyse the relationship between patient characteristics, mGPS, biological parameters, tumour site and overall and cancer-specific survival (Tables 1B and 2B), as well as the relationship between mGPS and survival (Figure 2A–F; Table 3). Cox proportional hazards model multivariate regression analysis (stratified by tumour site) was used to determine the relationship between patient characteristics, the mGPS and each biochemical parameter and survival (Tables 1C, D, 2C and D). *χ*^2^ (linear-by-linear) association was used to analyse the relationship between the mGPS, patient characteristics and biochemical parameters (Table 3). Owing to the number of statistical comparisons a *P*-value of <0.01 was considered significant. Analysis was performed using SPSS software (SPSS Inc., Chicago, IL, USA).

## Results

From Glasgow Inflammation Outcome Study of 223 303 patients originally described (Proctor *et al*, 2010), 21 669 patients were identified as having a diagnosis of cancer in the Scottish Cancer Registry and a blood sample taken between January 2000 and December 2006. There were 9608 patients in this group who had been sampled within 2 years before a diagnosis of cancer and included in the present study. The majority, 7516 (78%), were under 75 years of age. There were 5116 (53%) females and 4492 (47%) males. All patients had an identifiable postcode corresponding to a SIMD 2006 score with 15% of cases being from affluent areas (least deprived quintile of the Scottish population) and 38% being from deprived areas (most deprived quintile of the Scottish population). The minimum follow-up was 29 months and the maximum was 112 months (median 61 months for survivors).

The relationship between patient characteristics, mGPS, biochemical parameters, tumour site and mortality in patients with blood samples taken within 2 years before a diagnosis of cancer in the Glasgow Inflammation Outcome Study is shown in Table 1A. In total, 9608 patients were studied. On follow up, there were 6005 (63%) deaths of which 5122 (53%) were cancer deaths. The median time from blood sampling to diagnosis was 1.4 months.

**Figure 1:**
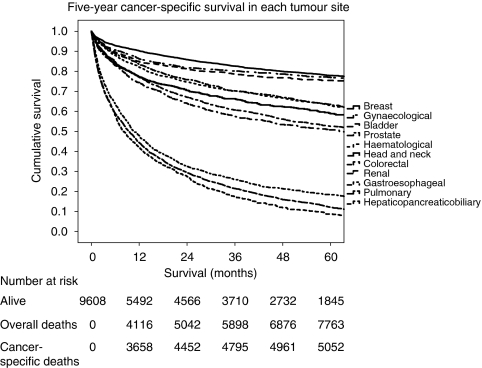
The relationship between tumour site and cancer-specific 5-year survival. Tumours from top to bottom: breast, gynaecological, bladder, prostate, haematological, head and neck, colorectal, renal, gastroesophageal, pulmonary and hepatopancreaticobiliary.

The relationship between patient characteristics, mGPS, biochemical parameters, tumour site and survival in patients with blood samples taken within 2 years before a diagnosis of cancer in the Glasgow Inflammation Outcome Study is shown in Table 1B and Figure 1. Increasing age, male gender and increasing deprivation were associated with reduced 5-year overall and cancer-specific survival (all *P*<0.001). A low albumin, an elevated mGPS, C-reactive protein, adjusted calcium, bilirubin, Alk phos, AST, ALT and GGT were associated with a reduced 5-year overall and cancer-specific survival (all *P*<0.001).

In the present cohort, the majority of patients with a low albumin concentration (*n*=2701) also had an elevated C-reactive protein concentration (*n*=2419, 90%). Few patients had a low albumin but a C-reactive protein concentration in the normal range (*n*=282). A low-albumin concentration alone was not significantly associated with cancer-specific survival in bladder (*P*=0.913), gynaecological (*P*=0.737), prostate (*P*=0.500), gastroesophageal (*P*=0.893), renal (*P*=0.945), colorectal (*P*=0.133), head and neck (*P*=0.740) and hepatopancreaticobiliary (*P*=0.209) cancers. In Cox multivariate regression analysis, the relationship between a mGPS of 2 and cancer-specific survival (hazard ratio (HR): 3.01, *P*<0.001) was stronger than that between C-reactive protein alone and cancer-specific survival (HR: 2.29, *P*<0.001). Therefore, in the remaining tables and figures only the mGPS was considered.

**Figure 2:**
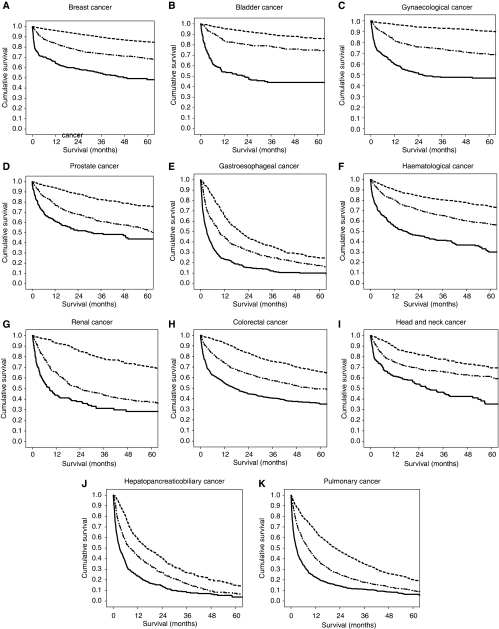
(**A**–**K**) The relationship between the mGPS 0 (top, small dash line), mGPS 1 (middle, large dash line) and mGPS 2 (bottom, solid line) and cancer specific survival (*P*<0.001) in each tumour site.

The relationship between the mGPS and cancer-specific survival in breast (*n*=1956), bladder (*n*=466), gynaecological (*n*=533), prostate (*n*=491), gastroesophageal (*n*=869), haematological (*n*=974), renal (*n*=424), colorectal (*n*=1065), head and neck (*n*=501), hepatopancreaticobiliary (*n*=605) and pulmonary (*n*=1724) cancers is shown in Figure 2A–K, respectively.

The relationship between the mGPS, biochemical parameters and survival in patients sampled within 2 years before cancer diagnosis in the Glasgow Inflammation Outcome Study, stratified by tumour site, is shown in Table 1C. On survival analysis, a raised mGPS, adjusted calcium, bilirubin, Alk phos, AST, ALT and GGT were all associated with increased overall and cancer-specific mortality independent of age, sex and deprivation (all *P*<0.001).

The relationship between patient characteristics, mGPS and survival in patients sampled within 2 years before cancer diagnosis in the Glasgow Inflammation Outcome Study is shown in Table 1D. On multivariate survival analysis, stratified by tumour site, increasing age and mGPS were associated with increased overall and cancer-specific mortality (all *P*<0.001). Patients in the most deprived quintile (5) had a reduced overall and cancer-specific survival (both *P*<0.01), but a significant linear relationship across deprivation categories was not observed.

The relationship between patient characteristics, mGPS, biochemical parameters, tumour site and mortality in patients with blood samples taken within 2 months before a diagnosis of cancer in the Glasgow Inflammation Outcome Study is shown in Table 2A. In total, 5397 patients were studied. On follow up, there were 3405 (63%) deaths, of which 2993 (56%) were cancer deaths. The median time from blood sampling to diagnosis was 0.4 month.

The relationship between patient characteristics, mGPS, biochemical parameters, tumour site and survival in patients with blood samples taken within 2 months before a diagnosis of cancer in the Glasgow Inflammation Outcome Study is shown in Table 2B. Increasing age, male gender and increasing deprivation were associated with reduced 5-year overall and cancer-specific survival (all *P*<0.001). An elevated mGPS, adjusted calcium, bilirubin, Alk phos, AST, ALT and GGT were associated with a reduced 5-year overall and cancer-specific survival (all *P*<0.001).

The relationship between the mGPS, biochemical parameters and survival in patients sampled within 2 months before cancer diagnosis in the Glasgow Inflammation Outcome Study, stratified by tumour site, is shown in Table 2C. On survival analysis, a raised mGPS, adjusted calcium, bilirubin, Alk phos, AST, ALT and GGT were all associated with increased overall and cancer-specific mortality independent of age, sex and deprivation (all *P*<0.001).

The relationship between patient characteristics, mGPS and survival in patients sampled within 2 months before cancer diagnosis in the Glasgow Inflammation Outcome Study is shown in Table 2D. On multivariate survival analysis, stratified by tumour site, increasing age and mGPS were associated with increased overall and cancer-specific mortality (all *P*<0.001). Patients in the most deprived quintiles (4 and 5) had a reduced overall and cancer-specific survival (both *P*<0.01), but a significant linear relationship across deprivation categories was not observed.

In the present cohort of patients sampled within 2 months prior to cancer diagnosis, only a limited number of patients had staging information available from the Scottish Cancer Registry. Tumour staging was available in 533 (39%) patients with breast cancer, 430 (76%) patients with colorectal cancer and 158 (13%) patients with pulmonary cancer. All other cancer groups had no staging available. Therefore, only in colorectal cancer was staging available in over 50% of patients. In these colorectal cancer patients, there were 30 Dukes A, 113 Dukes B, 131 Dukes C and 156 Dukes D, and 236 died of their cancer. When Dukes stage was included in the multivariate analysis, both Dukes stage (HR: 3.59, 95% confidence interval (CI): 2.95–4.39, *P*<0.001) and mGPS (HR: 1.49, 95% CI: 1.26–1.76, *P*<0.001) remained independently associated with survival.

The relationship between the mGPS, patient characteristics, biochemical parameters and overall and cancer-specific survival in patients with blood samples taken within 2 years before a diagnosis of cancer in the Glasgow Inflammation Outcome Study is shown in Table 3. An increasing mGPS was associated with increasing age, male gender, increasing adjusted calcium, bilirubin, Alk phos, AST, ALT and GGT (all *P*<0.001). An increasing mGPS was associated with a reduction in overall and cancer-specific survival (both *P*<0.001).

## Discussion

Previously, in large cohort studies, a number of biochemical parameters (other than C-reactive protein and albumin that compose the mGPS) including bilirubin (Temme *et al*, 2001), Alk phos (Tonelli *et al*, 2009), GGT (Kazemi-Shirazi *et al*, 2007) and calcium (Leifsson and Ahren, 1996), have been reported to predict overall and cancer-specific survival.

In the present study, the mGPS and the above biochemical parameters were shown to have prognostic value in all patients with a likely ongoing malignant process as well as those at the time of diagnosis. Moreover, an mGPS of 2 was associated with an ∼160% reduction in both overall and cancer-specific survival independent of tumour site. In contrast, an increase in adjusted calcium was associated with an approximate 130% reduction in both overall and cancer specific survival; an increase in bilirubin was associated with an approximate 50% reduction in both overall and cancer specific survival; an increase in Alk phos was associated with an approximate 110% reduction in both overall and cancer specific survival: an increase in AST was associated with an approximate 70% reduction in both overall and cancer specific survival; an increase in ALT was associated with an approximate 40% reduction in both overall and cancer specific survival and an increase in GGT was associated with an approximate 80% reduction in both overall and cancer specific survival. These results indicate that the mGPS and the biochemical parameters measured have prognostic significance in the tumour sites studied. Moreover, the results show that a raised mGPS is most closely associated with a reduction in both overall and cancer-specific survival, independent of tumour site.

The GPS was originally developed, from the combination of C-reactive protein and albumin, in a cohort of patients with advanced non-small cell lung cancer (Forrest *et al*, 2004). In this study, they were combined to give a score of 0 for both a normal C-reactive protein and albumin, 1 for either an abnormal C-reactive protein alone or albumin alone and 2 for both an abnormal C-reactive protein and albumin. It was clear from this analysis that a low albumin alone was uncommon, accounting for <10% of all observations, and raised the possibility that this was not associated with a reduced survival. When examined in a cohort of patients undergoing potentially curable resection for colorectal cancer (McMillan *et al*, 2008) the results showed that the relationship between an abnormal albumin alone and cancer-specific survival was similar to that of a normal albumin. Therefore, the GPS was modified (mGPS) to give a score of 1 only for an elevated C-reactive protein concentration. In the present study, in a much larger cohort, a low albumin alone was associated with poor survival in some tumours (breast, haematological and pulmonary) but not others (bladder, gynaecological, prostate, gastroesophageal, renal, colorectal, head and neck and hepatopancreaticobiliary). Therefore, the present results would indicate the greater consistency of the mGPS and suggest its general use rather than that of the GPS.

In the present study, we examined the relationship between the mGPS, biochemical parameters and survival in all patients with cancer as well as a subgroup of patients who were sampled within 2 months before a diagnosis of cancer. This was carried out with the premise that patients sampled within 2 years would have a mGPS associated with an ongoing malignant process and those sampled within 2 months would have a mGPS associated with their cancer diagnosis. With reference to the mGPS, the hazard ratios associated with survival remained consistent in both analyses and confirms the temporal utility of this inflammation-based prognostic score.

To date the mGPS has been shown to have prognostic value, independent of TNM stage, in lung, gastrointestinal and renal cancers (McMillan, 2008, 2009). In the present study, there was insufficient staging information available on all cancer groups, apart from colorectal, to demonstrate that the mGPS is universally prognostic independent of stage. However, the results of the present and previous studies (McMillan, 2009; Roxburgh and McMillan, 2010) would suggest that the mGPS might also have independent prognostic in other cancer types. Further detailed studies, including tumour stage, in breast, bladder, gynaecological, prostate, haematological, head and neck and hepatopancreaticobiliary cancers are required to confirm this hypothesis. Nevertheless, if this were to prove to be the case, then similar to the TNM staging system, the mGPS may be implemented universally in the assessment of cancer patients. Moreover, that they (TNM stage and mGPS) may be combined in a single staging system, which would not only account for tumour stage but also the host systemic inflammatory response.

The present cohort study has a number of limitations. The patients were selected on the basis that measurements of C-reactive protein, albumin and calcium had been performed and were therefore not necessarily representative of all cancer patients diagnosed and treated in the North Glasgow area. It is also recognised that patients with cancer may have concurrent morbidity causing a rise in their C-reactive protein and derangement of their albumin and other biochemical parameters.

In summary, the results of the present study indicate that, in a large patient cohort, the systemic inflammatory response, as evidenced by the mGPS, is common and that the mGPS is a powerful prognostic factor compared with other biochemical parameters, independent of tumour site in patients with cancer.

## Figures and Tables

**Table 1a:** The relationship between patient characteristics, mGPS, biochemical parameters, tumour site and mortality in patients sampled within 2 years before cancer diagnosis in the Glasgow Inflammation Outcome Study

	**Patients *n*=9608 (%)**	**All deaths *n*=6005**	**Cancer deaths *n*=5122**
*Age*			
<65 years	4577 (48)	2258	2056
65–74 years	2936 (30)	2041	1729
⩾75 years	2095 (22)	1706	1337
			
*Sex*			
Male	4492 (47)	3269	2794
Female	5116 (53)	2736	2328
			
*SIMD 2006*			
1 (least deprived)	1419 (15)	722	616
2	1198 (13)	631	543
3	1445 (15)	850	739
4	1858 (19)	1222	1038
5 (most deprived)	3688 (38)	2580	2186
			
*Tumour site*			
Breast	1956 (20)	452	328
Bladder	466 (5)	250	155
Gynaecological	533 (6)	298	256
Prostate	491 (5)	244	165
Gastroesophageal	869 (9)	772	719
Haematological	974 (10)	539	418
Renal	424 (5)	265	229
Colorectal	1065 (11)	673	571
Head and neck	501 (5)	316	220
Hepatopancreaticobiliary	605 (6)	563	536
Pulmonary	1724 (18)	1633	1525
			
*Inflammation-based prognostic score*			
mGPS			
0	3985 (42)	1647	1315
1	3204 (33)	2325	2039
2	2419 (25)	2033	1768
			
*Biochemical parameter*			
C-reactive protein			
⩽10 mg l^−1^	3985 (41)	1647	1315
>10 mg l^−1^	5623 (59)	4358	3807
Albumin			
>35 g l^−1^	6907 (72)	3765	3183
<35 g l^−1^	2701 (28)	2240	1939
Adjusted calcium			
<2.10 mmol l^−1^	329 (4)	229	177
2.10–2.60 mmol l^−1^	8856 (92)	5399	4601
>2.60 mmol l^−1^	423 (4)	377	344
Bilirubin			
<20 *μ*mol l^−1^	7936 (83)	4693	3978
>20 *μ*mol l^−1^	1342 (14)	1071	942
Alkaline phosphatase			
<80 U l^−1^	177 (2)	56	48
80–280U l^−1^	6846 (71)	3704	3068
>280 U l^−1^	2569 (27)	2236	1999
Aspartate transaminase			
<40 U l^−1^	7609 (18)	4439	3734
⩾40 U l^−1^	1678 (79)	1340	1206
Alanine transaminase			
<50 U l^−1^	6350 (66)	3588	3036
⩾50U l^−1^	992 (10)	741	677
*γ*-Glutamyl transferase			
M<70 U l^−1^; F<40 U l^−1^	6082 (63)	3336	2765
M⩾70 U l^−1^; F⩾40 U l^−1^	3264 (34)	2488	2212

Abbreviations: SIMD=Scottish Index of Multiple Deprivation; mGPS=modified Glasgow Prognostic Score.

**Table 1b:** The relationship between patient characteristics, mGPS, biochemical parameters, tumour site and survival in patients sampled within 2 years before cancer diagnosis in the Glasgow Inflammation Outcome Study

***n*=9608**	**5-year overall survival %**	***P*-value**	**5-year cancer-specific survival %**	***P*-value**
*Age*				
⩽65 years	50		54	
65–74 years	30		38	
⩾75 years	19	<0.001	30	<0.001
				
*Sex*				
Male	27		34	
Female	47	<0.001	53	<0.001
				
*SIMD*				
1 (least deprived)	50		55	
2	47		52	
3	41		46	
4	34		41	
5 (most deprived)	30	<0.001	37	<0.001
				
*Tumour site*				
Breast	77		83	
Bladder	57		64	
Gynaecological	44		52	
Prostate	50		64	
Gastroesophageal	11		13	
Haematological	46		54	
Renal	39		38	
Colorectal	37		43	
Head and neck	34		50	
Hepatopancreaticobiliary	6		7	
Pulmonary	5	<0.001	7	<0.001
				
*Inflammation-based prognostic score*				
mGPS				
0	59		65	
1	28		34	
2	15	<0.001	21	<0.001
				
*Biochemical parameter*				
C-reactive protein				
⩽10 mg l^−1^	59		65	
>10 mg l^−1^	23	<0.001	28	<0.001
Albumin				
⩾35 g l^−1^	46		52	
<35 g l^−1^	16	<0.001	22	<0.001
Adjusted calcium				
<2.10 mmol l^−1^	30		41	
2.10–2.60 mmol l^−1^	39		45	
>2.60 mmol l^−1^	10	<0.001	14	<0.001
Bilirubin				
<20 μmol l^−1^	41		47	
⩾20 μmol l^−1^	20	<0.001	25	<0.001
Alkaline phosphatase				
<80 U l^−1^	62		66	
80–280 U l^−1^	46		53	
>280 U l^−1^	13	<0.001	17	<0.001
Aspartate transaminase				
<40 U l^−1^	42		48	
⩾40 U l^−1^	20	<0.001	24	<0.001
Alanine transaminase				
<50 U l^−1^	43		50	
⩾50 U l^−1^	25	<0.001	28	<0.001
*γ*-Glutamyl transferase				
M<70 U l^−1^; F<40 U l^−1^	45		52	
M⩾70 U l^−1^; F⩾40 U l^−1^	23	<0.001	28	<0.001

Abbreviations: SIMD=Scottish Index of Multiple Deprivation; mGPS=modified Glasgow Prognostic Score.

**Table 1c:** The relationship between the mGPS, biochemical parameters and survival in patients sampled within 2 years before cancer diagnosis in the Glasgow Inflammation Outcome Study

	**Overall survival**		**Cancer-specific survival**	
	**HR**	***P*-value**	**HR**	***P*-value**
*mGPS*				
0	1	<0.001	1	<0.001
1	1.79	<0.001	1.92	<0.001
2	2.87	<0.001	3.01	<0.001
				
*Adjusted calcium*				
<2.10 mmol l^−1^	1.25	0.001	1.15	0.070
2.10–2.60 mmol l^−1^	1	<0.001	1	<0.001
>2.60 mmol l^−1^	2.30	<0.001	2.38	<0.001
				
*Bilirubin*				
<20 *μ*mol l^−1^	1	<0.001	1	<0.001
⩾20 *μ*mol l^−1^	1.53	<0.001	1.55	<0.001
				
*Alkaline phosphatase*				
<80 U l^−1^	0.71	0.012	0.74	0.037
80–280 U l^−1^	1	<0.001	1	<0.001
>280 U l^−1^	2.12	<0.001	2.19	<0.001
				
*Aspartate transaminase*				
<40 U l^−1^	1	<0.001	1	<0.001
⩾40 U l^−1^	1.66	<0.001	1.73	<0.001
				
*Alanine transaminase*				
<50 U l^−1^	1	<0.001	1	<0.001
⩾50U l^−1^	1.36	<0.001	1.39	<0.001
				
*γ-Glutamyl transferase*				
M<70 U l^−1^; F<40 U l^−1^	1	<0.001	1	<0.001
M⩾70 U l^−1^; F⩾40 U l^−1^	1.85	<0.001	1.85	<0.001

Abbreviations: HR=hazard ratio; mGPS=modified Glasgow Prognostic Score.

Adjusted for age, sex, deprivation and stratified by tumour site.

**Table 1d:** The relationship between patient characteristics, mGPS and survival in patients sampled within 2 years before cancer diagnosis in the Glasgow Inflammation Outcome Study

	**Overall survival**			**Cancer-specific survival**		
***n*=9608**	**HR**	**95 % CI**	***P*-value**	**HR**	**95 % CI**	***P*-value**
*Age*						
⩽65 years	1		<0.001	1		<0.001
65–74 years	1.35	1.26–1.43	<0.001	1.20	1.13–1.29	<0.001
⩾75 years	1.93	1.80–2.06	<0.001	1.59	1.48–1.70	<0.001
						
*Sex*						
Male	1		0.037	1		0.132
Female	0.94		0.037	0.95		0.132
						
*SIMD*						
1 (least deprived)	1		<0.001	1		0.003
2	1.02		0.684	1.02		0.773
3	1.09		0.090	1.09		0.136
4	1.16	1.06–1.27	0.002	1.12		0.025
5 (most deprived)	1.22	1.12–1.33	<0.001	1.17	1.06–1.28	0.001
						
*mGPS*						
0	1		<0.001	1		<0.001
1	1.79	1.68–1.91	<0.001	1.92	1.79–2.06	<0.001
2	2.87	2.68–3.08	<0.001	3.01	2.79–3.24	<0.001

Abbreviations: HR=hazard ratio; CI=confidence interval; SIMD=Scottish Index of Multiple Deprivation; mGPS=modified Glasgow Prognostic Score.

Multivariate analysis stratified by tumour site.

**Table 2a:** The relationship between patient characteristics, mGPS, biochemical parameters, tumour site and mortality in patients sampled within 2 months before cancer diagnosis in the Glasgow Inflammation Outcome Study

	**Patients *n*=5397 (%)**	**All deaths *n*=3405**	**Cancer deaths *n*=2993**
*Age*			
⩽65 years	2452 (46)	1192	1093
65–74 years	1686 (31)	1172	1035
⩾75 years	1259 (23)	1041	865
			
*Sex*			
Male	2315 (43)	1829	1620
Female	3082 (57)	1576	1373
			
*SIMD 2006*			
1 (least deprived)	802 (15)	392	335
2	685 (13)	344	304
3	766 (14)	429	388
4	974 (18)	648	572
5 (most deprived)	2170 (40)	1592	1394
			
*Tumour site*			
Breast	1383 (26)	235	166
Bladder	181 (3)	97	60
Gynaecological	188 (3)	129	108
Prostate	159 (3)	106	75
Gastroesophageal	456 (8)	400	371
Haematological	427 (8)	255	211
Renal	179 (3)	117	107
Colorectal	569 (11)	363	309
Head and neck	191 (4)	130	94
Hepatopancreaticobiliary	486 (9)	455	438
Pulmonary	1178 (22)	1118	1054
			
*Inflammation-based prognostic score*			
mGPS			
0	2243 (41)	890	743
1	1815 (34)	1385	1252
2	1339 (25)	1130	998
			
*Biochemical parameter*			
Adjusted calcium			
<2.10 mmol l^−1^	165 (3)	116	91
2.10–2.60 mmol l^−1^	4971 (92)	3058	2685
>2.60 mmol l^−1^	261 (5)	231	217
Bilirubin			
<20 *μ*mol l^−1^	4366 (81)	2558	2230
⩾20 *μ*mol l^−1^	855 (16)	720	655
Alkaline phosphatase			
<80 U l^−1^	98 (2)	28	25
80–280 U l^−1^	3788 (70)	2039	1746
>280 U l^−1^	1502 (30)	1333	1217
Aspartate transaminase			
<40 U l^−1^	4191 (78)	2409	2084
⩾40U l^−1^	1034 (19)	876	812
Alanine transaminase			
<50 U l^−1^	3740 (69)	2127	1847
⩾50 U l^−1^	636 (12)	514	483
*γ*-Glutamyl transferase			
M<70 U l^−1^; F<40 U l^−1^	3393 (63)	1841	1581
M⩾70 U l^−1^; F⩾40 U l^−1^	1867 (35)	1472	1338

Abbreviations: SIMD=Scottish Index of Multiple Deprivation; mGPS=modified Glasgow Prognostic Score.

**Table 2b:** The relationship between patient characteristics, mGPS, biochemical parameters, tumour site and survival in patients sampled within 2 months before cancer diagnosis in the Glasgow Inflammation Outcome Study

***n*=5397**	**5-year overall survival %**	***P*-value**	**5-year cancer-specific survival %**	***P*-value**
*Age*				
⩽65 years	51		54	
65–74 years	30		35	
⩾75 years	18	<0.001	26	<0.001
				
*Sex*				
Male	21		26	
Female	49	<0.001	54	<0.001
				
*SIMD*				
1 (least deprived)	52		57	
2	50		54	
3	43		47	
4	32		38	
5 (most deprived)	27	<0.001	32	<0.001
				
*Tumour site*				
Breast	83		87	
Bladder	48		63	
Gynaecological	33		39	
Prostate	32		46	
Gastroesophageal	12		15	
Haematological	40		47	
Renal	35		39	
Colorectal	37		43	
Head and neck	29		43	
Hepatopancreaticobiliary	5		6	
Pulmonary	5	<0.001	7	<0.001
				
*Inflammation-based prognostic score*				
mGPS				
0	60		65	
1	24		29	
2	15	<0.001	20	<0.001
				
*Biochemical parameter*				
Adjusted calcium				
<2.10 mmol l^−1^	29		39	
2.10–2.60 mmol l^−1^	38		43	
>2.60 mmol l^−1^	10	<0.001	13	<0.001
Bilirubin				
<20 μmol l^−1^	41		47	
⩾20 μmol l^−1^	15	<0.001	19	<0.001
Alkaline phosphatase				
<80 U l^−1^	68		69	
80–280 U l^−1^	46		52	
>280 U l^−1^	11	<0.001	14	<0.001
Aspartate transaminase				
<40 U l^−1^	42		48	
⩾40 U l^−1^	15	<0.001	18	<0.001
Alanine transaminase				
<50 U l^−1^	43		48	
⩾50 U l^−1^	19	<0.001	21	<0.001
*γ*-Glutamyl transferase				
M<70 U l^−1^; F<40 U l^−1^	46		51	
M⩾70 U l^−1^; F⩾40 U l^−1^	20	<0.001	25	<0.001

Abbreviations: SIMD=Scottish Index of Multiple Deprivation; mGPS=modified Glasgow Prognostic Score.

**Table 2c:** The relationship between the mGPS, biochemical parameters and survival in patients sampled within 2 months before cancer diagnosis in the Glasgow Inflammation Outcome Study

	**Overall survival**		**Cancer-specific survival**	
	**HR**	***P*-value**	**HR**	***P*-value**
*mGPS*				
0	1	<0.001	1	<0.001
1	1.67	<0.001	1.74	<0.001
2	2.36	<0.001	2.40	<0.001
				
*Adjusted calcium*				
<2.10 mmol l^−1^	1.17	0.107	1.07	0.530
2.10–2.60 mmol l^−1^	1	<0.001	1	<0.001
>2.60 mmol l^−1^	1.96	<0.001	2.04	<0.001
				
*Bilirubin*				
<20 *μ*mol l^−1^	1	<0.001	1	<0.001
⩾20 *μ*mol l^−1^	1.49	<0.001	1.49	<0.001
				
*Alkaline phosphatase*				
<80 U l^−1^	0.78	0.016	0.68	0.053
80–280 U l^−1^	1	<0.001	1	<0.001
>280 U l^−1^	1.93	<0.001	1.96	<0.001
				
*Aspartate transaminase*				
<40 U l^−1^	1	<0.001	1	<0.001
⩾40 U l^−1^	1.66	<0.001	1.71	<0.001
				
*Alanine transaminase*				
<50 U l^−1^	1	<0.001	1	<0.001
⩾50 U l^−1^	1.34	<0.001	1.37	<0.001
				
*γ-Glutamyl transferase*				
M<70 U l^−1^; F<40 U l^−1^	1	<0.001	1	<0.001
M⩾70 U l^−1^; F⩾40 U l^−1^	1.79	<0.001	1.79	<0.001

Abbreviations: HR=hazard ratio; mGPS=modified Glasgow Prognostic Score.

Adjusted for age, sex, deprivation and stratified by tumour site.

**Table 2d:** The relationship between patient characteristics, mGPS and survival in patients sampled within 2 months before cancer diagnosis in the Glasgow Inflammation Outcome Study

	**Overall survival**			**Cancer-specific survival**		
***n*=5397**	**HR**	**95 % CI**	***P*-value**	**HR**	**95 % CI**	***P*-value**
*Age*						
⩽65 years	1		<0.001	1		<0.001
65–74 years	1.31	1.20–1.42	<0.001	1.22	1.12–1.33	<0.001
⩾75 years	1.92	1.76–2.10	<0.001	1.70	1.55–1.87	<0.001
						
*Sex*						
Male	1		0.340	1		0.507
Female	0.96		0.340	0.97		0.507
						
*SIMD*						
1 (least deprived)	1		<0.001	1		<0.001
2	1.05		0.503	1.08		0.364
3	1.07		0.342	1.11		0.179
4	1.20	1.06–1.36	0.005	1.20	1.05–1.38	0.008
5 (most deprived)	1.30	1.16–1.45	<0.001	1.28	1.14–1.45	<0.001
						
*mGPS*						
0	1		<0.001	1		<0.001
1	1.67	1.53–1.83	<0.001	1.74	1.59–1.91	<0.001
2	2.36	2.15–2.59	<0.001	2.40	2.17–2.65	<0.001

Abbreviations: HR=hazard ratio; CI=confidence interval; SIMD=Scottish Index of Multiple Deprivation; mGPS=modified Glasgow Prognostic Score.

Multivariate analysis stratified by tumour site.

**Table 3:** The relationship between the mGPS, patient characteristics, biochemical parameters and survival in patients sampled within 2 years before cancer diagnosis in the Glasgow Inflammation Outcome Study

	**mGPS**			
	**0 *n* (%)**	**1 *n* (%)**	**2 *n* (%)**	***P*-value**
*Age*				
⩽65 years	2193 (55)	1464 (46)	920 (38)	
65–74 years	1091 (27)	1007 (31)	838 (35)	
⩾75 years	701 (18)	733 (23)	661 (27)	<0.001
				
*Sex*				
Male	1516 (38)	1672 (52)	1304 (54)	
Female	2469 (62)	1532 (48)	1115 (46)	<0.001
				
*SIMD 2006*				
1 (least deprived)	739 (19)	383 (12)	297 (12)	
2	592 (15)	342 (11)	264 (11)	
3	633 (16)	460 (14)	352 (15)	
4	727 (18)	613 (19)	518 (21)	
5 (most deprived)	1294 (32)	1406 (44)	988 (41)	<0.001
				
*Adjusted calcium*				
<2.10 mmol l^−1^	76 (2)	87 (3)	166 (7)	
2.10–2.60 mmol l^−1^	3835 (96)	2972 (93)	2049 (85)	
>2.60 mmol l^−1^	74 (2)	145 (4)	204 (8)	<0.001
				
*Bilirubin*				
<20 *μ*mol l^−1^	3564 (92)	2621 (85)	1751 (76)	
⩾20 *μ*mol l^−1^	324 (8)	466 (15)	552 (24)	<0.001
				
*Alkaline phosphatase*				
<80 U l^−1^	93 (2)	16 (0)	68 (3)	
80–280 U l^−1^	3428 (86)	2163 (68)	1255 (52)	
>280 U l^−1^	457 (12)	1020 (32)	1092 (45)	<0.001
				
*Aspartate transaminase*				
<40 U l^−1^	3491 (90)	2501 (81)	1617 (70)	
⩾40 U l^−1^	399 (10)	593 (19)	686 (30)	<0.001
				
*Alanine transaminase*				
<50 U l^−1^	3016 (91)	1982 (85)	1352 (79)	
⩾50 U l^−1^	301 (9)	337 (15)	354 (21)	<0.001
				
*γ-Glutamyl transferase*				
M<70 U l^−1^; F<40 U l^−1^	3071 (79)	1875 (60)	1136 (49)	
M⩾70 U l^−1^; F⩾40 U l^−1^	839 (21)	1242 (40)	1183 (51)	<0.001
				
Overall survival in months (mean, CI)	70 (68–72)	37 (36–39)	22 (21–24)	<0.001
Cancer-specific survival in months (mean, CI)	77 (75–79)	43 (41–45)	27 (26–29)	<0.001

Abbreviations: CI=confidence interval; SIMD=Scottish Index of Multiple Deprivation; mGPS=modified Glasgow Prognostic Score.
